# Single-cell RNA sequencing and spatial transcriptomics of esophageal squamous cell carcinoma with lymph node metastases

**DOI:** 10.1038/s12276-024-01369-x

**Published:** 2025-01-01

**Authors:** Wei Guo, Bolun Zhou, Lizhou Dou, Lei Guo, Yong Li, Jianjun Qin, Zhen Wang, Qilin Huai, Xuemin Xue, Yin Li, Jianming Ying, Qi Xue, Shugeng Gao, Jie He

**Affiliations:** 1 Department of Thoracic Surgery, National Cancer Center/National Clinical Research Center for Cancer/Cancer Hospital, Chinese Academy of Medical Sciences and Peking Union Medical College, Beijing, China; 2 Key Laboratory of Minimally Invasive Therapy Research for Lung Cancer, Chinese Academy of Medical Sciences, Beijing, China; 3 Department of Endoscopy, National Cancer Center/National Clinical Research Center for Cancer/Cancer Hospital, Chinese Academy of Medical Sciences and Peking Union Medical College, Beijing, China; 4 Department of Pathology, National Cancer Center/National Clinical Research Center for Cancer/Cancer Hospital, Chinese Academy of Medical Sciences and Peking Union Medical College, Beijing, China

**Keywords:** Oesophageal cancer, Metastasis

## Abstract

Esophageal squamous cell carcinoma (ESCC) patients often face a grim prognosis due to lymph node metastasis. However, a comprehensive understanding of the cellular and molecular characteristics of metastatic lymph nodes in ESCC remains elusive. In this study involving 12 metastatic ESCC patients, we employed single-cell sequencing, spatial transcriptomics (ST), and multiplex immunohistochemistry (mIHC) to explore the spatial and molecular attributes of primary tumor samples, adjacent tissues, metastatic and non-metastatic lymph nodes. The analysis of 161,333 cells revealed specific subclusters of epithelial cells that were significantly enriched in metastatic lymph nodes, suggesting pro-metastatic characteristics. Furthermore, stromal cells in the tumor microenvironment, including MMP3^+^IL24^+^ fibroblasts, APLN^+^ endothelial cells, and CXCL12^+^ pericytes, were implicated in ESCC metastasis through angiogenesis, collagen production, and inflammatory responses. Exhausted CD8^+^ T cells in a cycling status were notably prevalent in metastatic lymph nodes, indicating their potential role in facilitating metastasis. We identified distinct cell-cell communication networks and specific ligand-receptor pathways. Our findings were validated through a spatial transcriptome map and mIHC. This study enhances our comprehension of the cellular and molecular aspects of metastatic lymph nodes in ESCC patients, offering potential insights into novel therapeutic strategies for these individuals.

## Metastatic Lymph Nodes Reveal Insights in Esophageal Cancer

Esophageal cancer is a severe disease with low survival rates, especially a type called esophageal squamous cell carcinoma. When cancer spreads to lymph nodes, it worsens the prognosis, indicating a need for better treatments. Wei Guo and colleagues studied immune cell interactions in ESCC to discover new therapies. They examined 12 ESCC patients using advanced techniques like single-cell RNA sequencing and spatial transcriptomics. These methods analyze individual cell types and their locations, helping to understand the tumor microenvironment, which is vital for cancer spread. The study identified that certain cells, such as epithelial cells and fibroblasts, play a role in metastasis. Targeting these cells might enhance treatment options. The findings emphasize that understanding TME interactions is crucial for developing new therapies, and future research could focus on these interactions to improve ESCC patient outcomes.

This summary was initially drafted using artificial intelligence, then revised and fact-checked by the author.

## Introduction

As one of the most aggressive and lethal cancers, esophageal cancer caused approximately 0.54 million deaths all over the world in 2020^1,2^. The dominant subtype is esophageal squamous cell carcinoma (ESCC), accounting for more than 90% of esophageal cancer cases^3^. Patients with ESCC have an overall 5-year survival rate lower than 20% and the median survival time for patients with stage IV ESCC is less than one year^4,5^. Lymph node metastasis is one of the major factors contributing to the poor prognosis of ESCC patients and can further seed distant metastasis, which highlights the need to elucidate its processes to develop novel ESCC therapeutic approaches. Recent advancements in immune checkpoint inhibitor (ICI) therapy have shown encouraging outcomes in advanced ESCC patients, demonstrating the significant therapeutic potential of immunotherapy^6–8^. For instance, in a phase 3 clinical trial, overall survival rates were higher for metastatic ESCC patients receiving camrelizumab (a PD-1 inhibitor) and chemotherapy than those receiving placebo and chemotherapy^7^. Although some metastatic ESCC patients benefit from ICI therapy, a large subset of metastatic ESCC patients do not benefit from ICIs. Moreover, recent findings reveal that the efficacy of ICI therapy to ESCC is intricately related to the composition of immune infiltrating cells within tumor microenvironment (TME), suggesting the enormous potential of immune system for future cancer treatment^9,10^. Herein, thoroughly exploring the immune cell composition and intercellular interactions within the TME may help demonstrate the immune profiles and cellular processes of ESCC patients, providing novel insight into the development of innovative therapeutic approaches for metastatic ESCC.

Single-cell RNA sequencing (scRNA-seq) is a novel approach to unveiling cellular and molecular features of the TME at the single-cell level, revealing potential mechanisms underlying normal and abnormal biological processes in different cancers^11–13^. With the application of scRNA-seq technique, some pro-metastatic cells and metastasis-related contexture in cancer can be identified, facilitating the development of innovative and effective therapeutic approaches for metastatic cancer^14^. The scRNA-seq analysis has been undertaken on ESCC, revealing the intratumoral transcriptional heterogeneity and identifying some specific therapeutic biomarkers^15^. However, multicellular interactions of tumor ecosystem, distinct biological processes within the TME, as well as specific mechanisms underlying metastasis, remain largely unclear in metastatic ESCC. In addition, scRNA-seq technique is unable to evaluate the spatial characteristics and fully reveal the immune landscape within the TME. Advances in spatial transcriptomics (ST) provide another novel approach to reveal spatial characteristics and unravel heterogeneity of the TME, which could powerfully complement the use of scRNA-seq technique^16^. In our previous study, with the use of scRNA-seq and ST methods, differences in TME features between tumor samples from metastatic and non-metastatic ESCC patients were analyzed^17^. Since only investigating tumor samples is unable to fully reflect the distinctive immune profiles of metastatic ESCC, the research of metastatic lymph nodes is essential to advance our understanding of metastasis in ESCC, which may help the development of innovative treatment approaches.

In the present study, we intended to evaluate the immune microenvironment from tumor and adjacent nonmalignant tissues, as well as metastatic and non-metastatic lymph nodes. We have identified some metastasis-related cell subsets within the TME and investigated their specific functions in metastasis, intending to show dynamic geographical and cellular changes from non-metastatic to metastatic sites in ESCC. Our results enrich our understanding of immune features and cellular functions in metastatic ESCC, aiming to develop an innovative therapeutic intervention for this disease.

## Materials and methods

### Patient enrollment and sample collection

We have collected 29 samples from 12 patients with pathologically diagnosed ESCC who underwent surgery at the Department of Thoracic Surgery of the Cancer Hospital, Chinese Academy of Medical Sciences. All of the patients enrolled in our cohort have not received any treatment before the surgery. After the surgical removal, the fresh tumor and normal tissues were immediately collected for subsequent analysis. For scRNA-seq analysis, we have collected 12 ESCC samples, 6 adjacent normal tissues, 7 non-metastatic lymph nodes and 4 metastatic lymph nodes. Furthermore, the tumor sample of one patient with metastatic ESCC was processed for ST analysis. The clinicopathological data were presented in Supplementary Table S1. Our study was approved by the Ethics Committee of the National Cancer Center/Cancer Hospital (Approval number: 21/215-2886). We have obtained the written informed consent from all the patients enrolled in our cohort.

### Single-cell dissociation

Until processing, we surgically removed and kept tissues in MACS Tissue Storage Solution (Miltenyi Biotec). First, phosphate-buffered saline (PBS) was sued to wash the samples. Next, samples were minced into small pieces (approximately 1mm^3^) on ice and enzymatically digested with Tumor Dissociation Kit (Human) for 45 min at 37 °C, with agitation. Samples were sieved through a 70 µm cell strainer after digestion, and centrifuged at 300 g for 5 min. And the pelleted cells were suspended in red blood cell lysis buffer (Miltenyi Biotec) to lyse red blood cells for 5 min after the supernatant was removed. We re-suspended the cell pellets in PBS containing 0.04% BSA and re-filtered them with a 35μm cell strainer after washing with PBS containing 0.04% BSA. Using Countstar Fluorescence Cell Analyzer, we stained dissociated single cells with AO/PI for viability assessment.

### Single-cell sequencing

We generated the scRNA-Seq libraries with 10X Genomics Chromium Controller Instrument and Chromium Single Cell 3’ V3 Reagent Kits (10X Genomics, Pleasanton, CA). First, we concentrated cells to approximately 1000 cells/uL and loaded cells into each channel to generate single-cell Gel Bead-In-Emulsions (GEMs). Next, GEMs were broken and barcoded-cDNA was purified and amplified after the RT step. The amplified barcoded cDNA was fragmented, A-tailed, ligated with adaptors and index PCR amplified. We used the Qubit High Sensitivity DNA assay (Thermo Fisher Scientific) to quantify the final libraries and we used a High Sensitivity DNA chip on a Bioanalyzer 2200 (Agilent) to determine the size distribution of the libraries. All libraries were sequenced by illumina sequencer (Illumina, San Diego, CA) on a 150 bp paired-end run.

### Staining, permeabilization and reverse transcription for ST

We cut the cryosections at 10-μm thickness and mounted them onto the GEX arrays. Thermocycler Adaptor was used to mount the sections with the active surface facing up, which were then incubated for 1 min at 37 °C, fixed for 30 min with methyl alcohol in −20 °C, and finally stained with H&E (Eosin, Dako CS701, Hematoxylin Dako S3309, bluing buffer CS702). We used a Leica DMI8 whole-slide scanner to take the brightfield images at 10X resolution. Next, we used Visum spatial gene expression slide and Reagent Kit (10x Genomics, PN-1000184) to process visum spatial gene expression. We used Slide Cassette to create leakproof wells for adding reagents for each well. Then we added 70 μl Permeabilization enzyme and incubated it at 37 °C. We washed each well with 100 μl SSC and we added 75 μl reverse transcription Master Mix for cDNA Synthesis.

### cDNA library preparation for ST

We removed RT Master Mix from the wells at the end of first-strand synthesis. At room temperature, 75 μl 0.08 M KOH was added and incubated for 5 min. And we removed the KOH from wells and washed with 100 ul EB buffer. For second-strand synthesis, we added 75 μl Second Strand Mix to every well. A S1000TM Touch Thermal Cycler (Bio Rad) was used to perform cDNA amplication. We then used Visum spatial Library construction kit (10x Genomics, PN-1000184) to construct Visum spatial libraries based on the manufacture’s introduction. Finally, we used an Illumina Novaseq6000 sequencer to sequence the libraries with the sequencing depth of at least 100,000 reads per spot with pair-end 150 bp (PE150) reading strategy.

### Single-cell RNA statistical analysis

We performed the scRNA-seq data analysis with NovelBrain Cloud Analysis Platform. The fastp was applied with default parameter filtering the adaptor sequence and the low-quality reads were removed to achieve the clean data^18^. Using CellRanger v3.1.0, the feature-barcode matrices were obtained by aligning reads to the human genome (GRCh38 version 10X genomics). According to the mapped barcoded reads per cell of each sample, we conducted the down sample analysis among samples sequenced and finally achieved the aggregated matrix. We filtered cells containing more than 200 expressed genes and cells with mitochondria UMI rate below 20%. Based on the UMI counts of each sample and percent of mitochondria rate, we used Seurat package (version: 3.1.4, https://satijalab.org/seurat/) to normalize and regress cell and obtain the scaled data. According to the scaled data with top 2000 high variable genes, we performed PCA. And we used the top 10 principals for UMAP and tSNE construction. Based on the PCA top 10 principal, we obtained the unsupervised cell cluster result using graph-based cluster method. In addition, we used the FindAllMarkers function with Wilcoxon rank sum test algorithm to calculate the marker genes under the following criteria: 1. min.pct > 0.1; 2. *P* value < 0.05; 3. lnFC > 0.25. To screen for the detailed cell type, we select the clusters of the same cell type for re-tSNE analysis, graph-based clustering and marker analysis.

### Pseudo-time analysis

With DDR-Tree and default parameter, we used the Monocle2 (http://cole-trapnell-lab.github.io/monocle-release) to conduct the Single-Cell Trajectories analysis. We identified marker genes from the Seurat clustering result and the raw expression counts of the cells that passed filtering before performing Monocle analysis. Branch expression analysis modeling (BEAM), which was used for branch destiny determined gene analysis, was based on the pseudo-time analysis.

### Cell communication analysis

To enable a systematic analysis of cell-cell communication molecules, we applied cell communication analysis based on the CellChat (V1.1.3), a public repository of ligands, receptors and their interactions^19^. We quantized the cell-cell communication probability and inferred the statistically significant cellular communications using the CellChat with suggested parameters. The CellChat L-R interaction database provided the signaling pathway and the information flow scores were computed for each cell type. The likelihood of cellular interaction via a given pathway was characterized by the information flow scores. Information flow scores will be high in cells with high expression of a known ligand with cells having high expression of the corresponding receptor.

### SCENIC and QuSAGE analysis

As for SCENIC analysis, we used the Single-cell regulatory network inference and clustering (pySCENIC, v0.9.5) workflow to assess transcription factor regulation strength and we sued 20-thousand motifs database for RcisTarget and GRNboost^20^. We also conducted the QuSAGE (2.16.1) analysis to characterize the relative activation of a given gene set such as pathway activation^21^.

### CNV estimation

We used R package infercnv (v0.8.2) to identify somatic copy number variations and we used epithelial cells of adjacent tissues as the reference. Each cell for the extent of CNV signal was scored and the mean of squares of CNV values was defined across the genome. We then defined the putative malignant cells as those with CNV signal more than 0.05 and CNV correlation more than 0.5.

### GO and KEGG analysis

To elucidate the biological implications of differentially expressed genes and marker genes, we conducted Gene ontology (GO) analysis^22^. The GO annotations were downloaded from the Gene Ontology (http://www.geneontology.org/), UniProt (http://www.uniprot.org/) and NCBI (http://www.ncbi.nlm.nih.gov/). We performed the KEGG analysis to screen for significant pathway of differentially expressed genes and marker genes^23^. We used the Fisher’s exact test to select significant GO or KEGG categories and used FDR to correct the p-values.

### Multiplex immunofluorescence and image acquisition

Multiplex immunofluorescence staining was performed with 7-color fluorescence immunohistochemistry kit (PhenoVision Bio Co., Itd) following the company manual instruction. Tissue sections went through deparaffinization, rehydration, and were transferred into citrate buffer pH6.0 for antigenic repair. The sections were then washed and incubated with H_2_O_2_ and blocking reagents for 10 min, respectively. Primary antibodies (MMP3, POSTN, IL-24, SOF1, ABCC9, MYH11, EPCAM, Desmoplakin, TXNRD1, VEGFR2, Apelin, IGFBPS, SOX4, SOX11, CXCR4, SPARC, RGC32, CD3, CD4, CD8, PAN-CK, from OriGene, proteintech and Affinity, respectively) were applied on section for 30 min at RT, respectively. Bound primary antibodies were detected using PVB anti-Rb/Mm-HRP detection followed by PVB tyramide signal amplification fluorophore (PVB 520, PVB 570, PVB 620 or PVB 690) for 10 min. The slides were counterstained with spectral 4′,6-diamidino-2-phenylindole (PhenoVision Bio Co., Itd) and cover-slipped. The slides were scanned using the PhenoImage HT system (Akoya Biosciences) at 200X magnification.

### Statistical analysis

All statistical analyses were conducted using R (version 3.6.0) software. The Wilcoxon rank sum test was used to compare the difference between two groups. The threshold for statistical significance was *P* < 0.05.

## Results

### Characterization of ESCC revealed by scRNA-seq and spatial transcriptomics

**Fig. 1:**
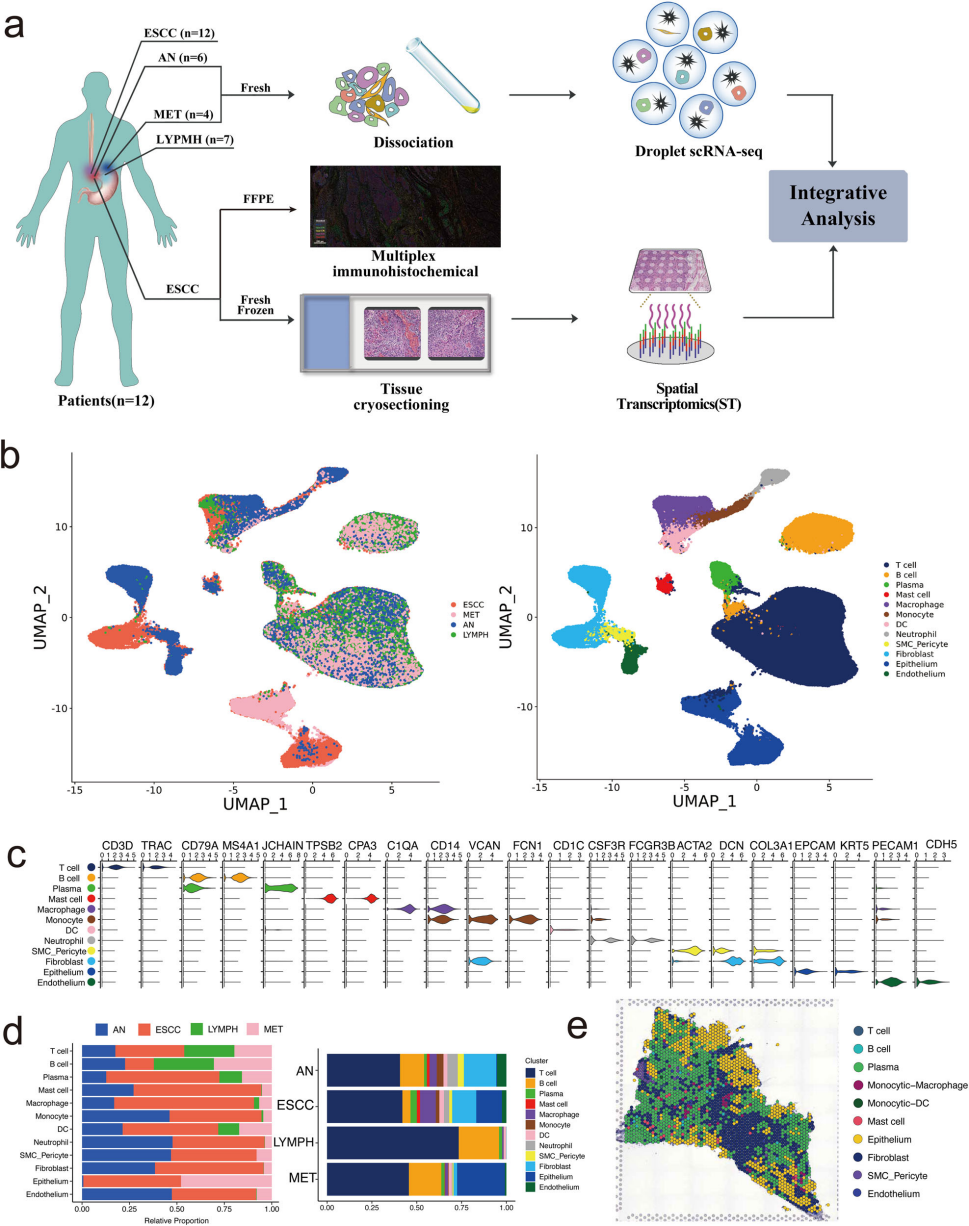
The scRNA-seq and ST profiles of esophageal squamous cell carcinoma (*n* = 12), adjacent normal tissues (*n* = 6), metastatic lymph nodes (*n* = 4) and non-metastatic lymph nodes (*n* = 7). **a** Schematic diagram depicting the design of this study. **b** UMAP plots of the 161,333 high-quality cells from all the 29 samples, with each color coded by either main annotated cell type (left) and corresponding sample type (right). **c** Violin plot depicting expression levels of the top genes across each main cell type. **d** Bar plots showing the proportion of each main cell type in different sample types. **e** The spot clustering of spatial features in the primary tumor sample obtained from one patient with metastatic ESCC. Each color represents one main cell type. The number of spots in the tumor sample is 2343.

To comprehensively elucidate the cellular characteristics of ESCC, we performed scRNA-seq analysis on 29 samples surgically obtained from 12 ESCC patients, including 12 tumor samples, 6 adjacent normal tissues, 4 metastatic lymph nodes and 7 non-metastatic lymph nodes (Fig. 1a). After eliminating putative cell doublets and dead or damaged cells during the filtration of the scRNA-seq data, a total of 161,333 high-quality cells were subjected to the following analysis, in which 61,928 cells originated from tumor samples, 29,518 cells from adjacent normal tissues, 49,966 cells from lymph nodes and 19,921 cells from metastatic lymph nodes. We have identified 12 main cell types according to well-known markers (Fig. 1b, c), including T cells (81,593), B cells (21,254), plasma cells (3845), mast cells (1660), macrophages (7403), monocytes (2458), dendritic cells (3261), neutrophils (3679), pericytes (2191), fibroblasts (14,248), epithelial cells (16,416) and endothelial cells (3325). Moreover, our results revealed that the fractions of T cells (Wilcoxon rank-sum test, LYMPH vs AN, *p* = 0.002; LYMPH vs ESCC, *p* = 0.007; LYMPH vs MET, *p* = 0.09) and B cells (Wilcoxon rank-sum test, LYMPH vs AN, *p* = 0.04; LYMPH vs ESCC, *p* = 0.0006; LYMPH vs MET, *p* = 0.2) were much higher in lymph nodes than in other samples, while the proportion of epithelial cells was the highest in metastatic lymph nodes (Fig. 1d). And the proportion of each cell subtype across different samples was shown in Supplementary Table S2. In addition, the spatial transcriptomic characterization of ESCC was revealed using the tumor sample from one patient with metastatic ESCC, and each main cell type was annotated on the ST sections (Fig. 1e). In general, we have revealed the cellular composition and spatial features of ESCC tissues, facilitating the subsequent analysis of metastasis-related cell subpopulation.

### Identification of epithelial cells with pro-metastatic features in metastatic ESCC

**Fig. 2:**
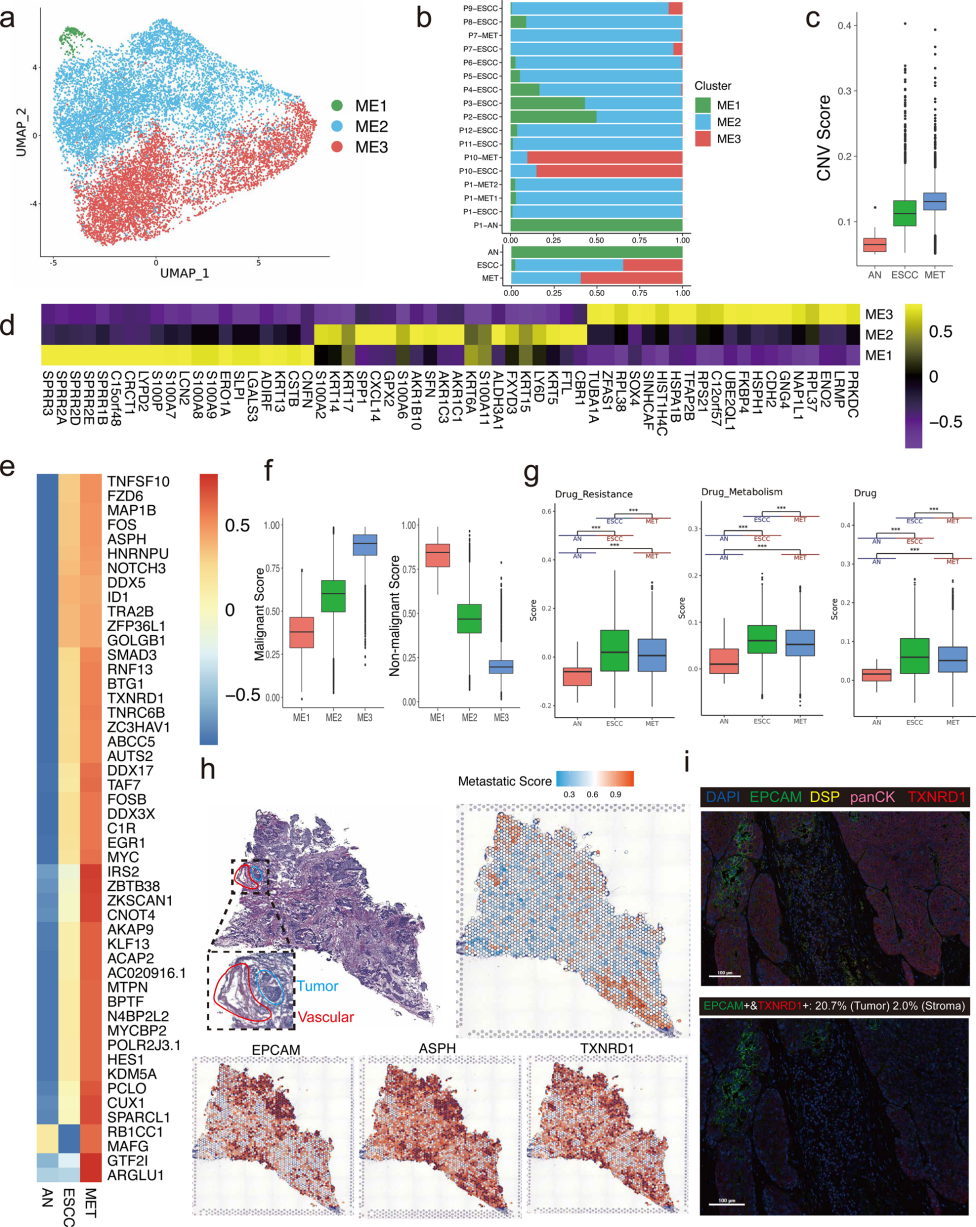
Exploration of pro-metastatic characteristics of epithelial cells in metastatic ESCC. **a** UMAP plots of the 16,416 high-quality cells from all the 21 samples, with each color coded by either cluster of epithelial cells. **b** The proportion of each epithelial cell cluster in different samples. **c** CNV scores of epithelial cells obtained from tumor samples, metastatic lymph nodes and adjacent tissues. **d** Heatmap depicting key marker genes for each subcluster of epithelial cells. **e** Heatmap showing relative expression levels of metastasis-related genes identified in metastatic ESCC. **f** Boxplot showing the malignant and non-malignant scores of each subcluster. **g** Specific GO functions enriched in epithelial cells obtained from metastatic lymph nodes in ESCC. **h** Representative H&E staining of tumor samples biopsied for spatial transcriptomics and spatial distribution of Metastatic Score, EPCAM, ASPH and TXNRD1 in the tumor site of one patient with metastatic lymph nodes. **i** The mIHC staining of EPCAM, DSP and TXNRD1 in the tumor site of one patient with metastatic lymph nodes. Scale bars: 100 μm.

We have identified 16,416 malignant epithelial cells collected from tumor samples, normal tissues and metastatic lymph nodes. A total of 3 epithelial cell subclusters were classified, most of which originated from tumor samples and metastatic lymph nodes (Fig. 2a, b). The majority of epithelial cells obtained from adjacent tissues were ME1 subcluster, implying the relatively low malignant potential of ME1 subcluster. ME2 (Wilcoxon rank-sum test, MET vs AN, *p* = 0.01; ESCC vs AN, *p* < 0.001; MET vs ESCC, *p* = 0.37) and ME3 (Wilcoxon rank-sum test, MET vs AN, *p* = 0.04; ESCC vs AN, *p* = 0.001; MET vs ESCC, *p* = 0.46) subclusters were predominantly collected from tumor samples and metastatic lymph nodes, indicating that ME2 and ME3 subclusters may have a high metastatic potential (Fig. 2b). Compared with epithelial cells obtained from adjacent tissues, epithelial cells obtained from metastatic lymph nodes have much higher CNV scores (Fig. 2c). The key marker genes of each subcluster were presented in Fig. 2d. In addition, our findings revealed that ME1 subcluster had the lowest malignant score and the highest non-malignant score among three subclusters, implying the low malignant potential of ME1 subcluster (Fig. 2f). To determine the pro-metastatic features in epithelial cells, we identified genes significantly upregulated in metastatic lymph nodes in two patients with metastatic lymph nodes samples (Supplementary Fig. S1a). We then intersected highly expressed genes of these two patients to identify genes associated with metastasis in ESCC (Supplementary Fig. S1b). We evaluated the expression levels of these genes in epithelial cells obtained from ESCC, adjacent tissues and metastatic lymph nodes, and subsequently selected genes highly expressed in metastatic lymph nodes as the pro-metastatic genes (Fig. 2e). We then conducted survival analysis to screen for genes correlated with poor prognosis. The Kaplan–Merier curves of the GSE53625 cohorts indicated that high expression levels of *TXNRD1* were related to unfavorable prognosis (Supplementary Fig. S1c). Compared with functions in metastatic lymph nodes, our findings revealed drug metabolism and drug resistance were highly enriched in primary tumor (Fig. 2g). At the histological level, tumor regions were determined in the H&E image and ST feature plots (Fig. 2h). Specific metastasis-related regions were identified according to pro-metastatic features, and vessels were found around the metastasis-related regions, indicating that the recruitment of new blood vessels may be essential for ESCC metastasis. The mIHC images revealed that marker genes of epithelial cells (EPCAM, DSP and TXNRD1) were highly expressed in tumor regions, especially in the metastasis-related regions (Fig. 2i). The selected genes were validated by using spatial analysis and mIHC, and the above results showed that TXNRD1 was highly expressed in the pro-metastatic region.

### The mCAF1 (MMP3^+^IL24^+^) facilitated ESCC metastasis via degrading extracellular matrix and promoting angiogenesis

**Fig. 3:**
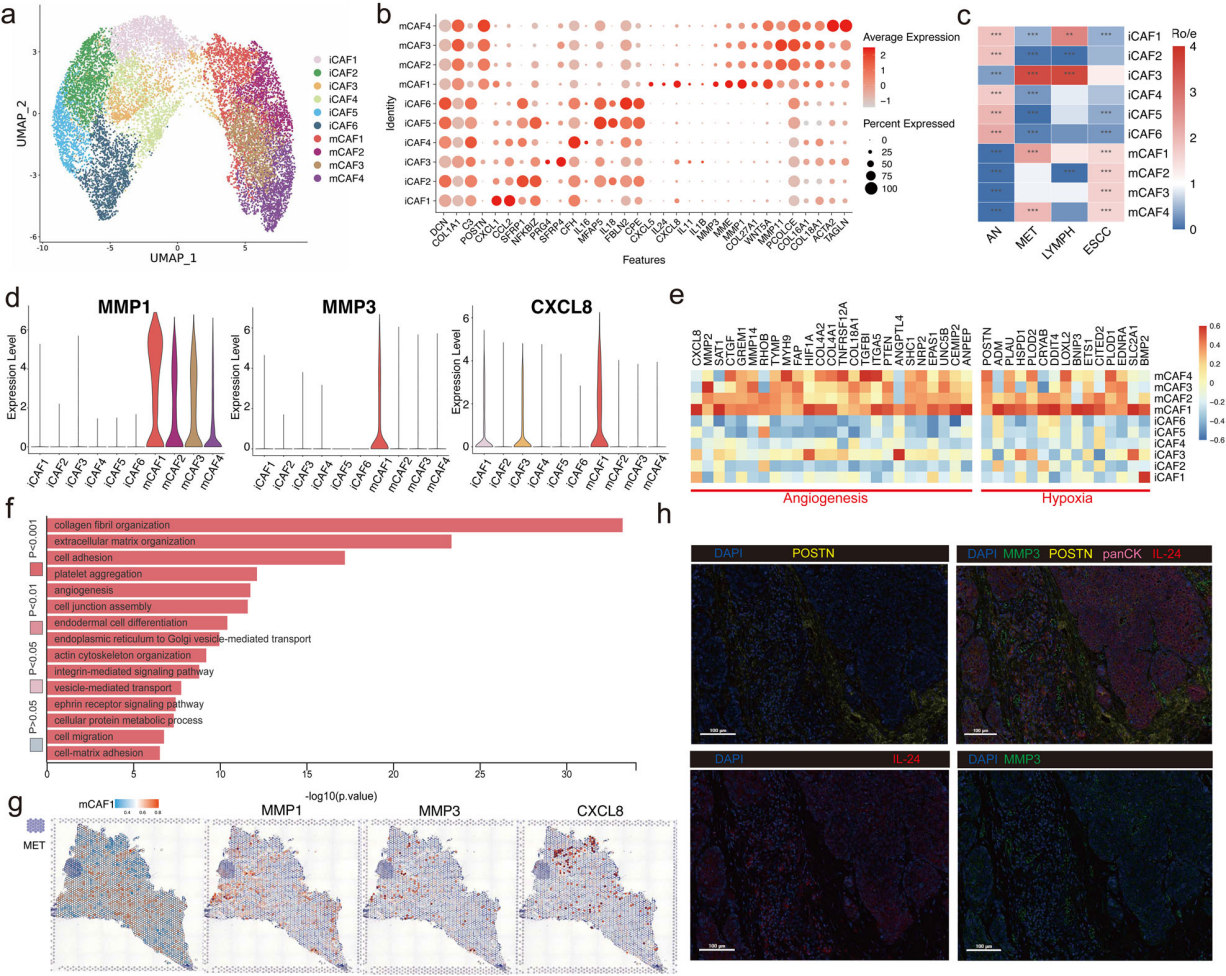
Identification of cancer-associated fibroblasts correlated with collagen reduction and angiogenesis in metastatic ESCC. **a** UMAP plots showing 14,248 high-quality cells from all the 29 samples, with each color coded by either main annotated cell type. **b** Bubble plot showing key marker genes for each subcluster of fibroblasts. **c** Heatmap comparing the proportion of fibroblasts among tumor samples, adjacent normal tissue, metastatic lymph nodes and non-metastatic lymph nodes. *P < 0.05; **P < 0.01; ***P < 0.001. **d** Violin plots showing the expression levels of markers correlated with collagen reduction and angiogenesis in different subpopulations. **e** Heatmap depicting the expression levels of angiogenesis correlated genes in different fibroblast subpopulations. **f** Bar plot showing the GO functions enriched in mCAF1 subpopulation. **g** Spatial distribution of mCAF1 subpopulation and MMP3, MMP1 and CXCL8 in the tumor site of one patient with metastatic lymph nodes. **h** The mIHC staining of MMP3, POSTN and IL-24 in the tumor site of one patient with metastatic lymph nodes. Scale bars: 100 μm.

The surrounding tumor microenvironment, particularly the extracellular matrix (ECM), may also play a salient role in shaping cancer progression and promoting metastasis^24^. We then investigated the subpopulations of cancer-associated fibroblasts (CAFs). According to prior findings and our study, we have grouped 14,248 CAFs into two major subclusters: iCAF and mCAF (Fig. 3a). The iCAF subcluster was mainly enriched in growth factors, including the WNT and NF-kB pathways (*SFRP1* and *NFKBIZ*), and some chemokine markers (*CXCL1* and *CCL2*), whereas mCAF subcluster had significantly accumulated *POSTN* genes (Fig. 3b). A total of four mCAF subclusters and six iCAF subclusters were then subsequently identified, and we found distinct expression profiles of different subclusters (Fig. 3b).

Our findings revealed that some CAF subclusters were simultaneously enriched in metastatic lymph nodes and primary tumor sites, such as mCAF1 (MMP3^+^IL24^+^) and mCAF4, suggesting that these subclusters may serve as key roles in facilitating metastasis in ESCC (Fig. 3c). To further investigate how these CAF subclusters facilitate metastasis, we identified that expression levels of *MMP1* and *MMP3* were significantly higher in mCAF1 than in other subclusters (Fig. 3d). MMP3 could degrade the basement membrane and ECM, providing conditions for tumorigenesis and metastasis^25^. Similar to the function of *MMP3*, the dysfunction of p53 could elevate *MMP1* expression and subsequently enhance the metastasis of lung adenocarcinoma^26^. The expression level of *CXCL8* was also predominantly higher in mCAF1 than in other subclusters (Fig. 3d). As one of the most potent chemokines, *CXCL8* normally serves as a key factor in responding to infection. Recently, *CXCL8* has also been recognized as an important gene promoting angiogenesis, which may provide a necessary basis for facilitating cancer progression and metastasis^27^. Furthermore, we found antiangiogenic genes (such as *HIF1A*) and hypoxia-related genes (such as *ADM*), were highly expressed in mCAF1 (Fig. 3e). The functional analysis also revealed that angiogenesis was predominantly enriched in mCAF1 (Fig. 3f).

The regions of mCAF1 and correlated marker genes were also validated using ST and mIHC with samples from a patient with metastatic ESCC, revealing that mCAF1 was predominantly abundant in the stromal regions of the TME (Fig. 3g, h). The mCAF1 subcluster was also surrounding the pro-metastatic region in the ST feature plot, indicating the involvement of mCAF1 subcluster in the metastasis of ESCC (Fig. 3g). Herein, we proposed that the degradation of ECM and angiogenesis in the tumor microenvironment promoted by mCAF1 may contribute to metastasis in ESCC.

### SOX4 and SOX11 drive APLN^+^ endothelial cells towards pro-metastatic phenotype via regenerative angiogenesis and collagen production

**Fig. 4:**
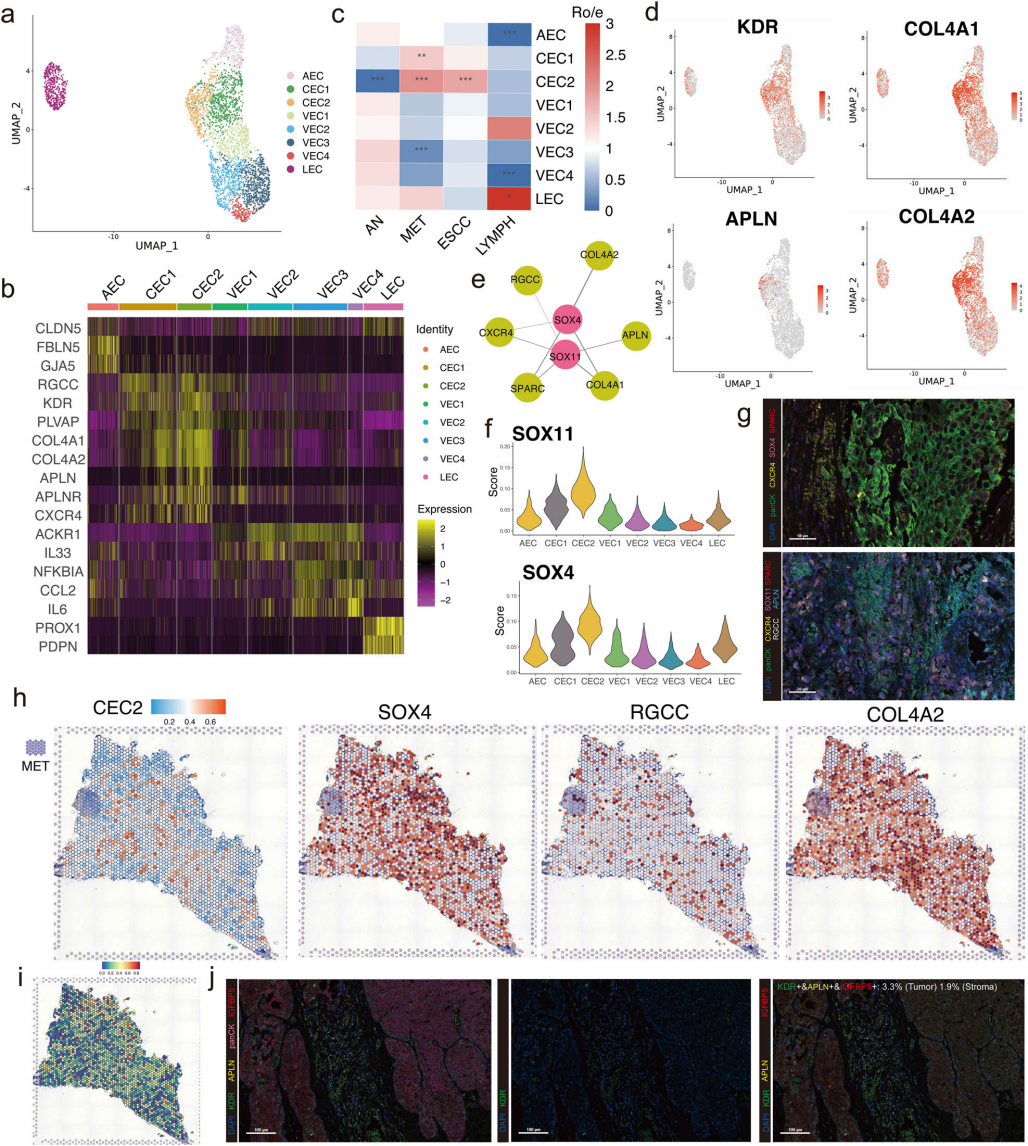
Endothelial cells serving important roles in the metastasis of ESCC. **a** UMAP plots of 3325 endothelial cells grouped into 8 subpopulations. **b** Heatmap showing the expression levels of marker genes in different endothelial cell subpopulations. **c** Heatmap comparing the proportion of endothelial cells among tumor samples, adjacent normal tissue, metastatic lymph nodes and non-metastatic lymph nodes. *P < 0.05; **P < 0.01; ***P < 0.001. **d** UMAP plots depicting the expression levels of KDR, APLN, COL4A1 and COL4A2 in endothelial cells. **e** The representative regulatory network of SOX4 and SOX11 revealed by SCENIC analysis. **f** The activity scores of SOX4 and SOX11 in different endothelial cell subpopulations. **g** The mIHC staining of SOX4, CXCR4, SPARC, SOX11, RGCC and APLN in the tumor site of one patient with metastatic lymph nodes. Scale bars: 50 μm. **h** Spatial distribution of CEC2 subpopulation and SOX4, RGCC and COL4A2 in the tumor site of one patient with metastatic lymph nodes. **i** The deconvolution analysis showing the distribution of CEC2 subpopulation in the tumor site of one patient with metastatic lymph nodes. **j** The mIHC staining of KDR, APLN, IGFBP5 in the tumor site of one patient with metastatic lymph nodes. Scale bars: 100 μm. AEC arterial endothelial cells, CEC capillary endothelial cells, VEC vascular endothelial cells, LEC lymphatic endothelial cells.

Endothelial cells are important components of the ECM, which normally serve as critical regulators in angiogenesis, extracellular matrix organization and immunomodulation during cancer progression^28^. A total of 3325 endothelial cells grouped into 4 clusters have been identified, including 1 arterial endothelial cell (AEC) subcluster, 2 capillary endothelial cell (CEC) subclusters, 4 vascular endothelial cell (VEC) subclusters and 1 lymphatic endothelial cell (LEC) subcluster (Fig. 4a). Our findings have revealed that CEC subclusters had significantly accumulated genes correlated with the function of capillaries, such as *RGCC* and *PLVAP* (Fig. 4b, Supplementary Fig. S2a). We found that CEC subclusters, especially CEC2, were predominantly abundant in primary tumor samples and metastatic lymph nodes, indicating that CEC2 may serve as a key role in ESCC metastasis (Fig. 4c).

To further explore the role of CEC2 in metastatic ESCC, we identified that *APLN* and *APLNR* were highly expressed in CEC2, also known as APLN^+^ endothelial cells (Fig. 4d, Supplementary Fig. S2a). *APLN* is usually regarded as an essential biomarker associated with regenerative angiogenesis, which may have the potential to switch endothelial cells from a mature state to a proliferative state^29^. Moreover, *COL4A1* and *COL4A2* were highly expressed in CEC subclusters, especially in APLN^+^ endothelial cells, indicating that these subclusters served an important role in producing collagen (Fig. 4d). Collagen adhesion is a complicated mechanism occurring during cancer invasion and metastasis, which may be the potential function of APLN^+^ endothelial cells. In addition, COL4A1 could activate FAK-Src signaling pathway and then facilitate cancer progression and metastasis^30^. The functional analysis revealed that focal adhesion and some important pathways in tumor metastasis were enriched in APLN^+^ endothelial cells, such as PI3K-Akt signaling pathway and Notch signaling pathway (Supplementary Fig. S2b).

We then conducted SCENIC to identify upstream transcription factors of APLN^+^ endothelial cells, which may drive APLN^+^ endothelial cells to pro-metastatic phenotype^20^. Of note, *SOX4* and *SOX11*, were among the transcription factors most significantly upregulated in APLN^+^ endothelial cells (Fig. 4f, Supplementary Fig. S2c). Our findings have revealed that SOX4 mostly regulated genes correlated with collagen production (such as *CXCR4*, *COL4A1* and *COL4A2*), and SOX11 regulated genes related to regenerative angiogenesis (*RGCC* and *APLN*), which further facilitated the differentiation of endothelial cells towards pro-metastatic phenotype in the TME (Fig. 4e). The regulation networks of SOX4 and SOX11 were validated by the mIHC plots (Fig. 4g). Furthermore, ST feature plots and results of deconvolution analysis showed that APLN^+^ endothelial cells surrounded the pro-metastatic region of tumor cells, and the expression levels of key marker genes were highly expressed (Fig. 4h–i). The mIHC plots were used to further validate the results, showing the pro-metastatic characteristics of APLN^+^ endothelial cells in ESCC (Fig. 4j). In addition, we found that some legends, such as POSTN, TNFSF11 and TNFSF12, were highly activated in metastatic lymph nodes, indicating their potential role in facilitating metastasis in ESCC (Supplementary Fig. S2d).

### Discontinuous blood vessels formed by smooth muscle cells and inflammatory responses activated by PC1 (CXCL12^+^) promoted ESCC metastasis

**Fig. 5:**
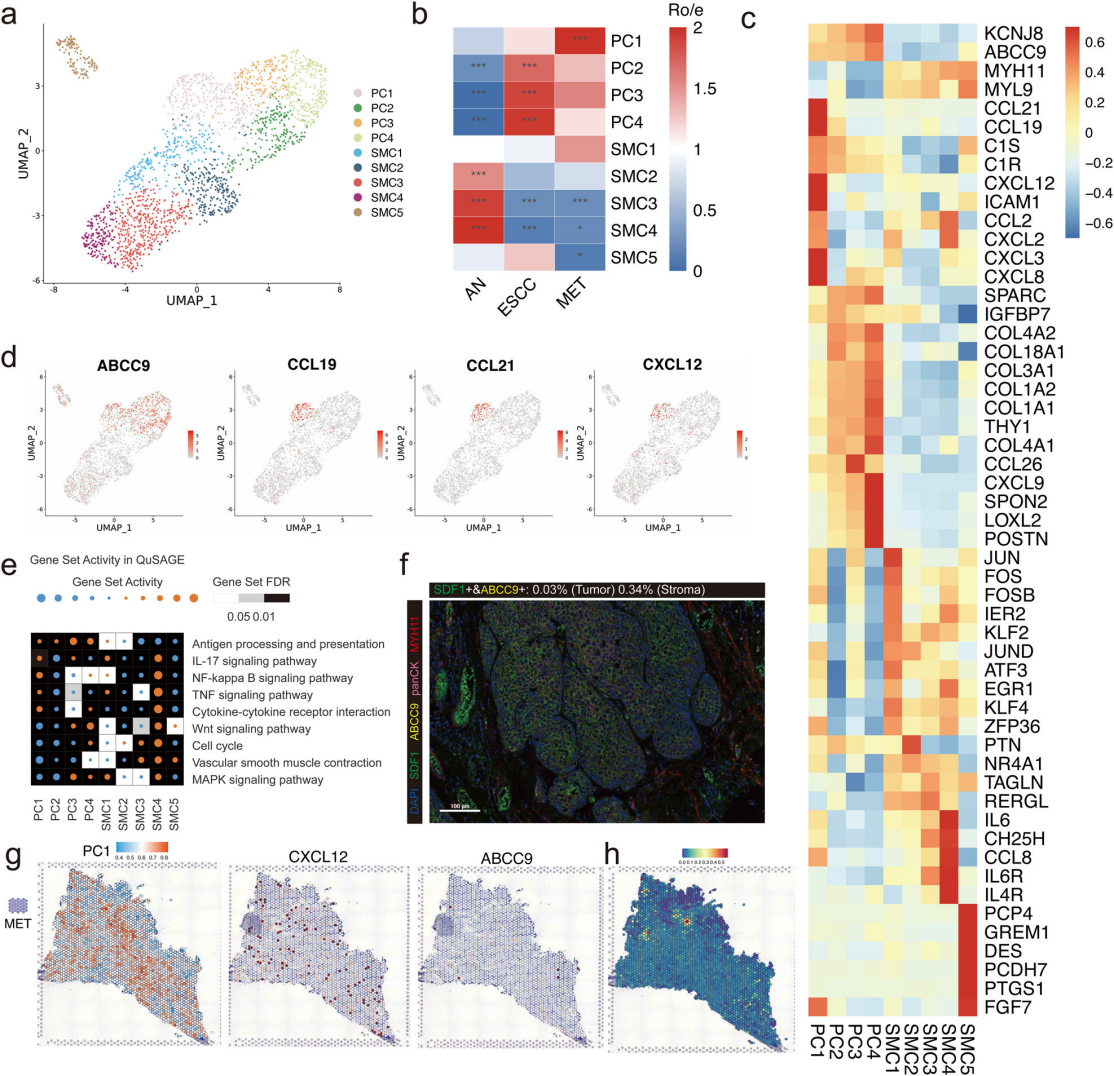
Metastatic lymph nodes of ESCC are composed of specific pericytes and smooth muscle cells (SMCs). **a** UMAP plots of 2191 pericytes and SMCs grouped into 9 clusters. **b** Heatmap comparing the proportion of pericytes and SMCs among esophageal squamous cell carcinoma, adjacent normal tissue, metastatic lymph nodes and non-metastatic lymph nodes. *P < 0.05; **P < 0.01; ***P < 0.001. **c** Heatmap showing the selected marker genes of each pericyte and SMC subcluster. **d** UMAP plots depicting the expression levels of ABCC9, CCL19, CCL21 and CXCL12 in pericytes and SMCs. **e** Heatmap showing the gene set QuSAGE enrichment scores for different pericyte and SMC subcluster subclusters. **f** The mIHC staining of SDF1, ABCC9, MYH11 in the tumor site of one patient with metastatic lymph nodes. Scale bars: 100 μm. **g** Spatial distribution of PC1, CXCL12 and ABCC9 in the tumor site of one patient with metastatic lymph nodes. **h** The deconvolution analysis showing the distribution of PC1 subpopulation in the tumor site of one patient with metastatic lymph nodes.

We further explore the role of other stromal cells in the TME, such as smooth muscle cells (SMCs) and pericytes (PCs). A total of 2191 cells were classified into 4 PC subpopulations and 5 SMC subpopulations in ESCC (Fig. 5a). Our findings revealed that the proportion of most SMC subclusters was significantly decreased in metastatic lymph nodes, whereas PC1 subcluster was predominantly abundant (Fig. 5b). SMCs are critical components of blood vessels and the depletion of SMCs may lead to the formation of an incomplete circulatory system, providing an ideal environment for metastasis^31^. For pericytes, the proportion of PC1 was significantly higher in metastatic lymph nodes, implying the potential role of PC1 subcluster in the metastasis of ESCC (Fig. 5b). As a chemokine with a crucial function in cancer progression, CXCL12 could bind to CXCR4 and induce multiple signaling pathways related to angiogenesis, cancer progression and metastasis^32^. Our findings revealed that *CXCL12* is highly expressed in PC1 subcluster, indicating that PC1 may activate specific inflammatory pathways in metastatic ESCC (Fig. 5c, d). Next, we conducted QuSAGE analysis and identified some pathways significantly enriched in PC1 subcluster, such as IL-17 signaling pathway, NF-κB signaling pathway and TNF signaling pathway (Fig. 5e). The ST feature plots, mIHC plots and results of deconvolution analysis were used to validate our findings (Fig. 5f–h).

### Exhausted CD8^+^ T cells in cycling status may be essential components of the pro-metastatic tumor microenvironment

**Fig. 6:**
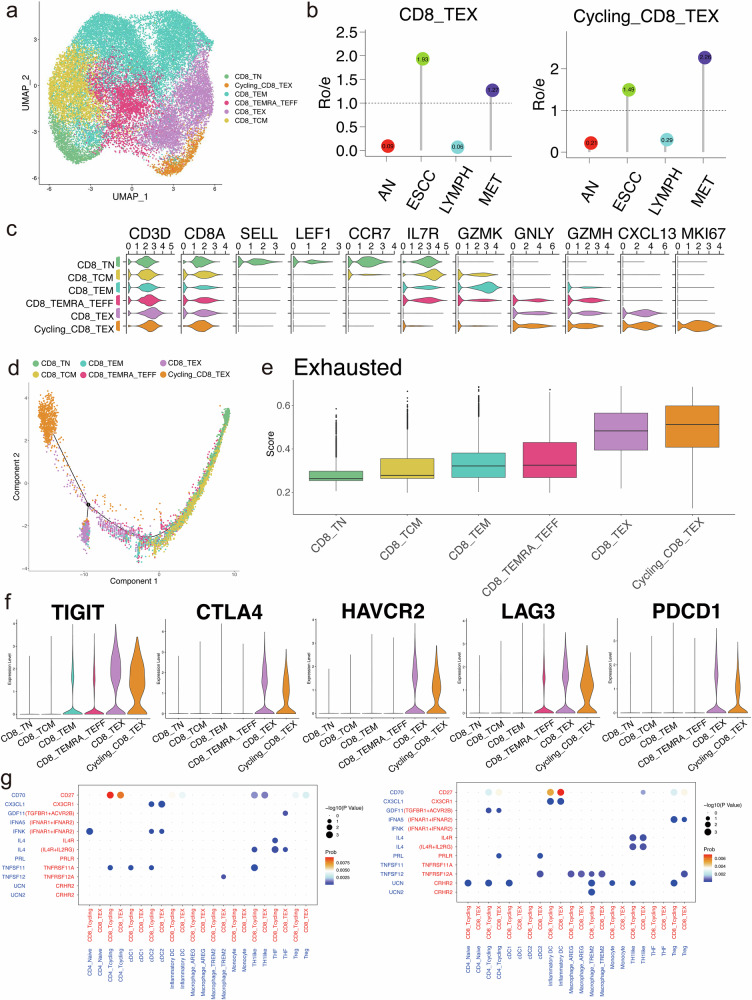
Exhausted CD8^+^ T cells in cycling status in the pro-metastasis environment for ESCC metastasis. **a** UMAP plots of CD8^+^ T cells into 6 clusters. **b** The relative proportion of exhausted CD8^+^ T cells (TEX) and exhausted CD8^+^ T cells in cycling status (Cycling-TEX) among esophageal squamous cell carcinoma, adjacent normal tissue, metastatic lymph nodes and non-metastatic lymph nodes. **c** Violin plot showing the expression levels of key marker genes of each CD8^+^ T cell subcluster. **d** Potential trajectory of differentiation from naïve CD8^+^ T cells (CD8-TN) into exhausted CD8^+^ T cells in cycling status (Cycling-TEX) inferred by analysis with Monocle 2. **e** Comparison of exhausted scores of each subcluster of CD8^+^ T cells. **f** Violin plots showing key marker genes of exhausted CD8^+^ T cells. **g** Bubble plots comparing ligand-receptor signaling pathways of immune cells between normal lymph nodes (left) and metastatic lymph nodes (right) in metastatic ESCC.

A total of 25,816 CD8^+^ T cells were identified and classified into six subclusters (Fig. 6a). Our findings revealed that cycling and exhausted CD8^+^ T cells were relatively abundant in tumor samples and metastatic lymph nodes (Fig. 6b, Supplementary Fig. S3a). We have divided exhausted CD8^+^ T cells (CD8-TEX) into two subgroups, exhausted CD8^+^ T cells in normal status (CXCL13^+^MKI67^-^) and exhausted CD8^+^ T cells in cycling status (CXCL13^+^MKI67^+^) (Fig. 6c, Supplementary Fig. S3b). To further explore the dynamic transitional process of CD8^+^ T cells in metastatic ESCC, we constructed a pseudotime map of CD8^+^ T cell state trajectory via using the monocle2^33^. The trajectory was stated with naïve CD8^+^ T cells (CD8-TN), via central memory CD8^+^ T cells (CD8-TCM), effector memory CD8^+^ T cells (CD8-TEM) and effector CD8^+^ T cells (CD8-TEMRA-TEFF) as the transitional state between CD8-TN and CD8-TEX, and terminally reached a final differentiate state of Cycling-CD8-TEX (Fig. 6d, Supplementary Fig. S3c, d). We evaluated the levels of exhaustion of each CD8^+^ T cell subcluster, implying that Cycling-CD8-TEX and CD8-TEX had the highest scores (Fig. 6e). Immune checkpoint inhibitors (ICIs) are regarded as essential targets for cancer treatment, and some inhibitory receptors are correlated with T-cell exhaustion^34^. We have analyzed the expression levels of 5 ICIs among different CD8^+^ T cell subclusters, which revealed that ICIs were highly expressed in Cycling-CD8-TEX and CD8-TEX, indicating the exhaustion status of these two subclusters (Fig. 6f, Supplementary Fig. S3e). To further explore the distinctive cellular interactions of lymph node metastasis, we analyzed differential cell-cell communications in normal and metastatic lymph nodes (Fig. 6g). Compared with normal lymph nodes, CD70-CD27 and IL4-IL4R interactions were significantly identified, which may activate CD8-TEX and subsequently suppress the tumor microenvironment.

### Type-I interferons secreted by CD4^+^ Tregs and TREM2^+^ macrophages may play an important role in ESCC metastasis

We have identified 51,001 CD4^+^ T cells for five subclusters, with 36,644 naïve T cells accounting for the majority of all CD4^+^ T cells (Supplementary Fig. S4a, b). Our findings showed that CD4^+^ Tregs were more abundant in tumor samples and metastatic lymph nodes than in adjacent tissues and normal lymph nodes, implying the potential role of CD4^+^ Tregs in facilitating lymph node metastasis in ESCC (Supplementary Fig. S4c). Generally, Tregs play a critical role in preventing autoimmunity and inhibiting antitumor immune responses^35^. To investigate the potential role of CD4^+^ Tregs in lymph node metastases, we identified the upregulated genes in CD4^+^ Tregs from metastatic lymph nodes, including *IFI27*, *IL2RA* and *IL2RB* (Supplementary Fig. S4d). Furthermore, we have conducted the functional analysis of the CD4^+^ Tregs in the metastatic lymph nodes, and type I interferon signaling pathway was highly enriched (Supplementary Fig. S4e). Type-I interferons are central coordinators of the immune system, which could serve as pro-tumor or anti-tumor factors^36^. In addition, *IFI27* is an important gene in type-I interferons signaling pathway and could promote malignant progression and angiogenesis in ESCC, which may be one of the key regulators in ESCC metastasis^37^. We also validated the regions of CD4^+^ Tregs using ST, deconvolution analysis and mIHC, showing that the enrichment of T cells was not affected by differences in cell viability among cell types (Supplementary Fig. S4f–h).

Studies have revealed that myeloid cells play a critical role in TME^38^. We have identified 8 subclusters of myeloid cells, including monocytes, 3 types of macrophages (Macrophage-resident, TREM2^+^ macrophages, AREG^+^ macrophage) and 4 types of dendritic cells (cDC1, cDC2, pDC and inflammatory-DC) (Supplementary Fig. S5a). The key marker genes of each myeloid cell subcluster were enriched in Supplementary Fig. S5b. Among all the subclusters, the relative proportion of TREM2^+^ macrophages was much higher in tumor samples and metastatic lymph nodes, indicating that TREM2^+^ macrophages might serve as an important factor in ESCC metastasis (Supplementary Fig. S5c). A recent study has shown that TREM2^+^ macrophages could suppress the function of CD8^+^ T cells in hepatocellular carcinoma and may serve as a pro-tumor factor^39^. In addition, TREM2^+^ macrophages are highly related to angiogenesis, lipid metabolism and inflammation (Supplementary Fig. S5d). Phagocytosis and antigen-presenting activity are critical anti-tumor functions of macrophages, which could reduce carcinogenesis and progression^40^. Our findings revealed that TREM2^+^ macrophages from metastatic lymph nodes had a minimal role in phagocytosis and antigen processing & presentation among all sample types, suggesting the potential mechanism by which TREM2^+^ macrophages promote metastasis in ESCC (Supplementary Fig. S5e–g). Similar to CD4^+^ Tregs, we have identified some genes correlated with type-I interferons signaling pathway highly expressed in TREM2^+^ macrophages of metastatic lymph nodes (Supplementary Fig. S5h). Given the important regulatory role of the type-I interferon signaling pathway in the function of macrophages, we propose that TREM2^+^ macrophages may facilitate ESCC metastasis through type-I interferon signaling pathway^41^.

## Discussion

Recent studies have demonstrated the distinctive characteristics of the immune cells of ESCC^10,15,42^. However, an in-depth investigation of the microenvironment of the metastatic and non-metastatic lymph nodes in ESCC at single-cell level has not been conducted. Here, we used 29 samples from 12 ESCC patients, including tumor samples, adjacent normal tissues, metastatic and non-metastatic lymph nodes, to comprehensively illustrate the cellular and molecular features in the microenvironment of metastatic ESCC at single-cell level. We built a spatial transcriptome atlas and used mIHC images to further explore the mechanisms of ESCC metastasis and validate the findings of scRNA-seq. The integration of scRNA-seq and ST helped provide the foundation for the development and application of novel therapeutic strategies for metastatic ESCC.

As metastasis remains a significant challenge in cancer prevention and treatment, it is imperative to uncover the cellular and molecular characteristics of the TME to gain insights into lymph node metastases in ESCC. Given the inherent heterogeneity of tumor cells, the task of identifying distinctive epithelial cell subpopulations correlated with metastasis is exceptionally challenging. Previous studies have identified particular epithelial cell subclusters that facilitate tumor metastasis in some other cancers, whereas key subclusters promoting metastasis have not been identified in ESCC^15,43^. According to our study, epithelial cells exhibiting pro-metastatic features are abundant in metastatic lymph nodes, which may possess significant metastatic potential in ESCC. Zhang et al. have dissected unique expression programs of malignant epithelial cells in ESCC, such as the stress responses program that included the activation of genes (*FOS*, *EGR1* and *JUN*) in response to cellular stimuli, revealing some highly expressed genes similar to epithelial cells with pro-metastatic features^44^. For example, a recent study showed that EGR1 may promote cancer metastasis via facilitating angiogenesis^45^. Our findings also showed that drug resistance and drug metabolism functions were not significantly enriched in metastatic lymph nodes, indicating the pro-metastatic characteristics of these epithelial cell subpopulations^46^. At the histological level, we have identified metastasis-related regions based on pro-metastatic features. Notably, blood vessels were observed surrounding these specific regions, implying that the recruitment of new blood vessels may play a crucial role in ESCC metastasis.

Stromal cells within the TME, such as fibroblasts and endothelial cells, play pivotal roles in both tumorigenesis and metastasis, and they also have the capacity to modulate the TME^47^. In general, tumor cells migrate along ECM fibers toward blood vessels, implying the importance of the TME in metastasis. Our findings revealed that mCAF1 (MMP3^+^IL24^+^) was one of the important subpopulations in metastatic ESCC, exhibiting pro-metastatic characteristics via matrix degradation and angiogenesis. MMP3 can degrade the basement membrane and ECM, subsequently reducing adhesion in cells and providing conditions conducive to the migration of tumor cells, which serves as a crucial facilitator of metastasis in ESCC^25,48^. In addition, mCAF1 may promote ESCC metastasis by facilitating angiogenesis, with the angiogenesis pathway being notably enriched in mCAF1. For example, *CXCL8* exhibits high expression levels in mCAF1, and it is widely recognized as a crucial gene promoting angiogenesis, potentially facilitating cancer progression and metastasis^27^. The spatial transcriptome atlas also showed that mCAF1 was found surrounding the metastasis-related region, further corroborating our findings.

Endothelial cells have the tube formation ability and play a crucial role in angiogenesis, a process closely correlated with cancer progression and metastasis^49^. According to our study, a capillary endothelial cell subcluster (APLN^+^) was significantly abundant in both primary tumor and metastatic lymph nodes, indicating the pro-metastatic ability of APLN^+^ endothelial cells. APLN has the capacity to direct the differentiation of endothelial cells and assist in vascular repair following injury^50^. A recent study has demonstrated that inhibition of *APLN* could profoundly suppress angiogenesis-dependent tumor growth, suggesting the pivotal role of APLN in remodeling the TME and facilitating angiogenesis^51^. Considering that vessel function improved upon targeting APLN, we propose that APLN^+^ endothelial cells may remain in an immature state and could potentially form incomplete blood vessels, thereby enabling tumor cells’ access to the circulatory system and facilitating metastasis in ESCC^52^. Moreover, as an important constituent of ECM, collagen may serve as a crucial driver of tumor metastasis^53^. We found that *COL4A1* and *COL4A2* were highly expressed in APLN^+^ endothelial cells, indicating that collagen may also play a significant role in ESCC metastasis. We also explored the potential upstream mechanisms of collagen production and some transcription factors may be vital regulators. According to a recent study, SOX4 and SOX11 could promote breast cancer metastasis^54,55^. Our findings have revealed significant upregulation of *SOX4* and *SOX11* in APLN^+^ endothelial cells, and showed their potential role in regulating collagen production and angiogenesis. This implies that SOX4 and SOX11 may contribute to the differentiation of endothelial cells toward a pro-metastatic phenotype.

The microenvironment of lymph nodes differs from primary tumor sites, with lymph nodes containing more immune cells that play a crucial role in tumor immunity^56^. Immunosuppression is a hallmark of the TME, potentially promoting tumor growth and metastasis^9^. However, no study has previously reported the molecular and cellular features of metastatic lymph nodes in ESCC. Normally, the cytotoxicity of CD8^+^ T cells is crucial to suppress tumor growth and metastasis, and CD8^+^ T cell exhaustion was an essential promoter for tumor metastasis^57^. According to our findings, we identified exhausted CD8^+^ T cells in cycling status (CXCL13^+^MKI67^+^), which were relatively abundant in metastatic lymph nodes, implying that this particular subpopulation may serve as a potential therapeutic biomarker for metastatic ESCC. Moreover, TREM2^+^ macrophages, as another important type of immune cells, were highly abundant in metastatic lymph nodes. Our results showed that type-I interferons signaling pathway was highly enriched in TREM2^+^ macrophages of metastatic lymph nodes. Considering the significant regulatory role of the type-I interferon signaling pathway in macrophages, we propose that TREM2^+^ macrophages may promote ESCC metastasis through this important pathway^41^. Similar to our findings, Gong et al. have revealed that the suppressive activity of Tregs can be enhanced through the CD70-CD27 interaction, implying the crucial role of CD70-CD27 interaction in immunosuppressive microenvironment and the metastasis^58^. IL4 signaling pathway served as an important role in tumor progression and metastasis^59^. Our findings revealed that IL4-IL4R interaction may regulate CD8^+^ exhausted T cells to promote ESCC metastasis, providing novel therapeutic insights into cancer treatment.

Our study included several limitations. Although 29 samples were collected for subsequent scRNA-seq analyses, the limited number of some specific specimens may lead to bias because tumor samples were very heterogeneous. For example, the number of metastatic lymph nodes was relatively smaller than that of tumor samples. Furthermore, for some patients with metastatic ESCC, metastatic lymph nodes were only collected for pathological diagnosis and were not available for our analyses, resulting in different types of samples not being entirely matched in all patients. However, our study has presented a comprehensive cellular landscape of metastatic ESCC, and provided a valuable basis for in-depth investigation of the initiation and progression of ESCC with lymph node metastasis.

In conclusion, we have offered distinctive perspectives and critical information for the spatial patterns and distinct cellular components involved in ESCC metastasis. We have identified particular epithelial cells exhibiting pro-metastatic characteristics in ESCC, potentially serving as the primary initiators of metastasis. Furthermore, more attention is needed on stromal cells, including fibroblasts, endothelial cells, smooth muscle cells and pericytes, as they may play pivotal roles in promoting metastasis via regulating angiogenesis and influencing the formation of the extracellular matrix. In addition, it has recently come to light that various immune cells within the tumor microenvironment actively contribute to metastasis in ESCC. The recently identified pro-metastatic cellular components and the distinctive intercellular communications within the tumor microenvironment will be crucial for the development of effective therapeutic strategies for metastatic ESCC.

## Supplementary information

Supplementary information

Supplementary Table1

Supplementary Table2

## Data Availability

The data that supporting the findings of this study can be obtained from the corresponding author upon reasonable request.
