# Supplementary material for: Single-cell RNA sequencing and spatial transcriptomics of esophageal squamous cell carcinoma with lymph node metastases
Source: Exp Mol Med. 2025 Jan 1;57(1):59–71. doi: 10.1038/s12276-024-01369-x (PMC11799171; doi:10.1038/s12276-024-01369-x)
Supplement: Supplementary file 1 — Supplementary information [file 12276_2024_1369_MOESM1_ESM.pdf]

Supplementary Materials

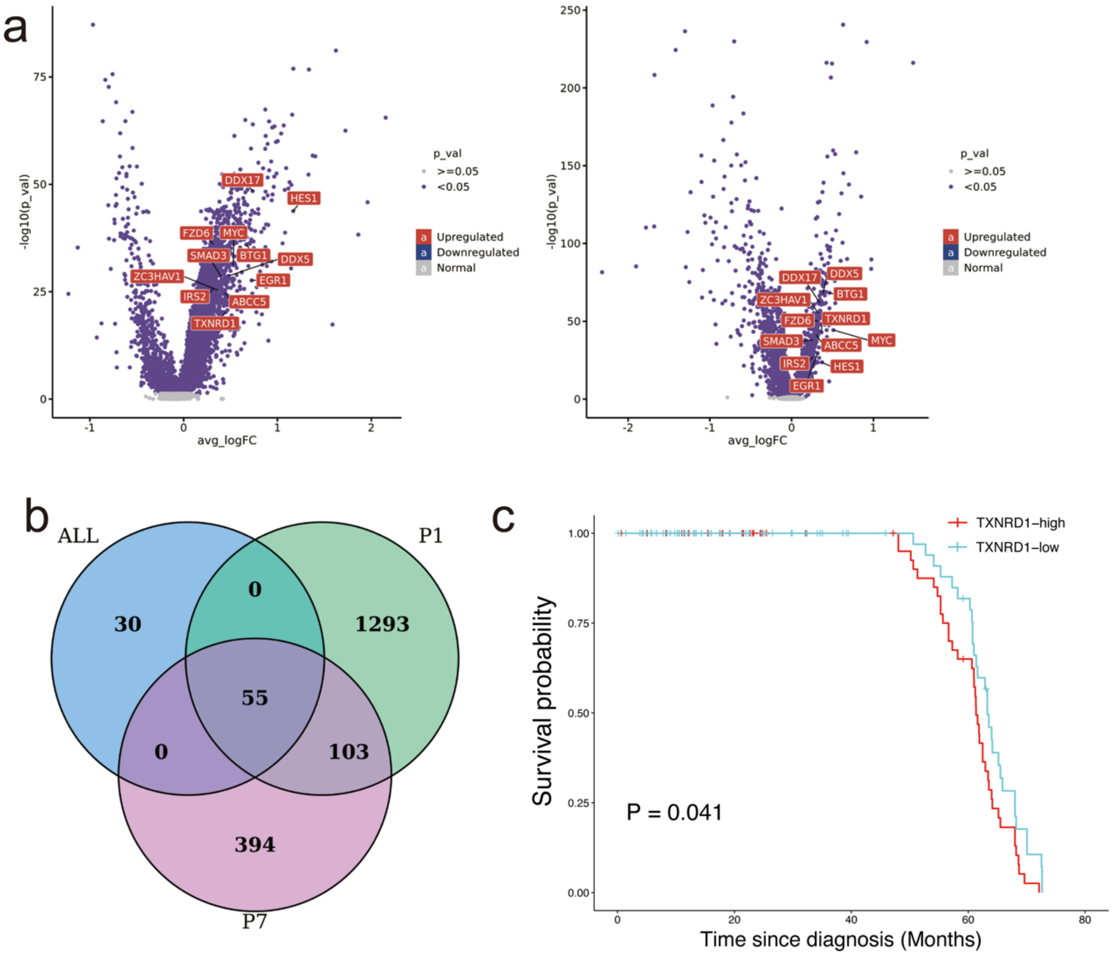

Supplementary Fig.1 Identifying pro-metastatic genes of epithelial cells in metastatic ESCC. a. Volcano plots showing the differentially expressed genes between metastatic lymph nodes and tumor samples in two patients (P1 and P7) with metastatic lymph node biopsies. b. Venn plot showing the intersection of the differentially expressed genes in P1 and P7. c. Kapan-Merier curves showing the prognostic value of TXNRD1 in the GSE53625 cohort.

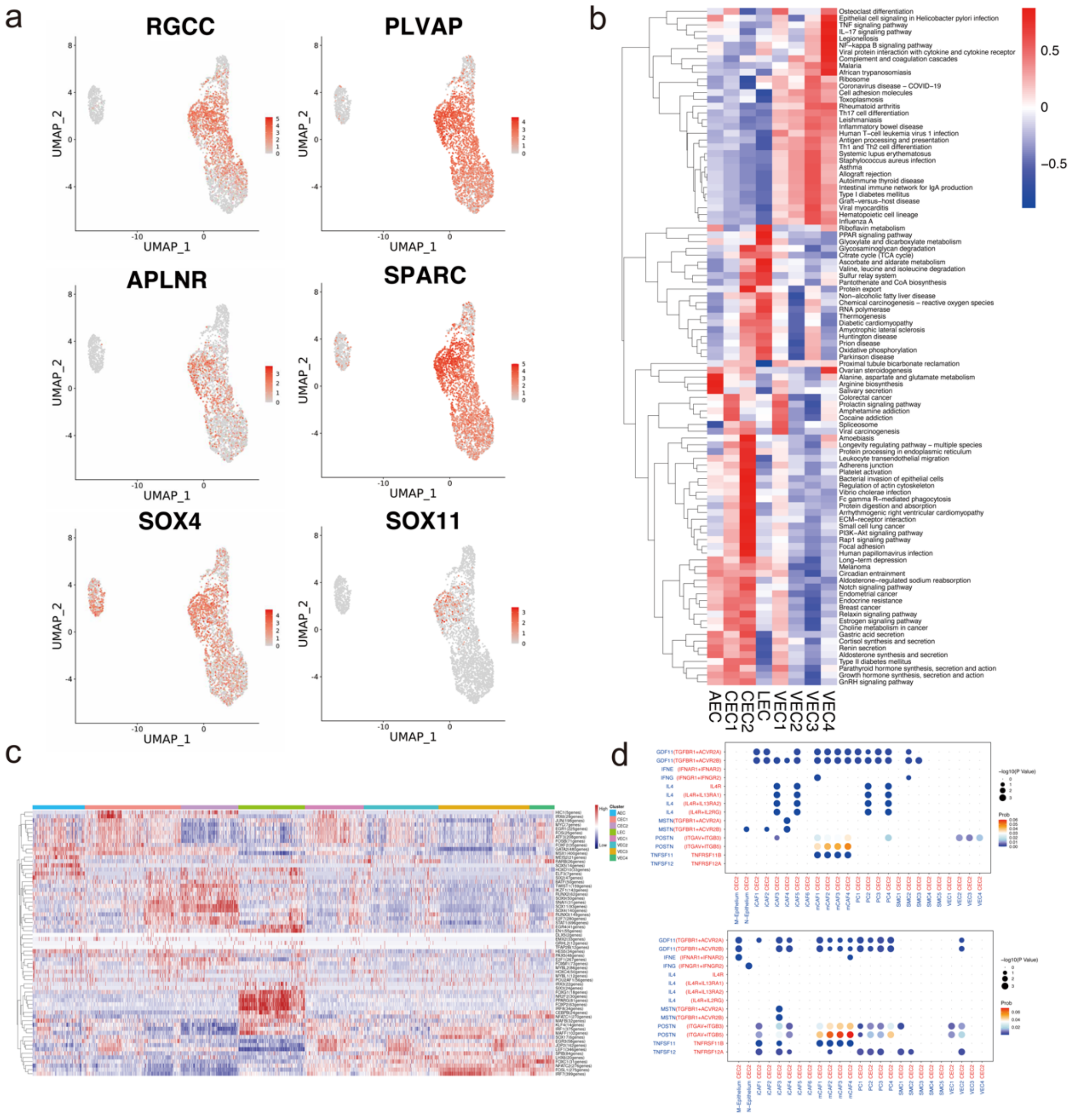

Supplementary Fig.2 Transcription factors and functional analysis of different endothelial cell subpopulations in ESCC. a. UMAP plots depicting the expression levels of key marker genes in endothelial cells. b. Pathways enriched in different endothelial cell subpopulations. c. SCENIC analysis identifying potential upstream transcription factors of each endothelial cell subpopulation. d. Bubble plots comparing ligand-receptor signaling pathways of cells between primary tumor (upper) and metastatic lymph nodes (lower) in metastatic ESCC.

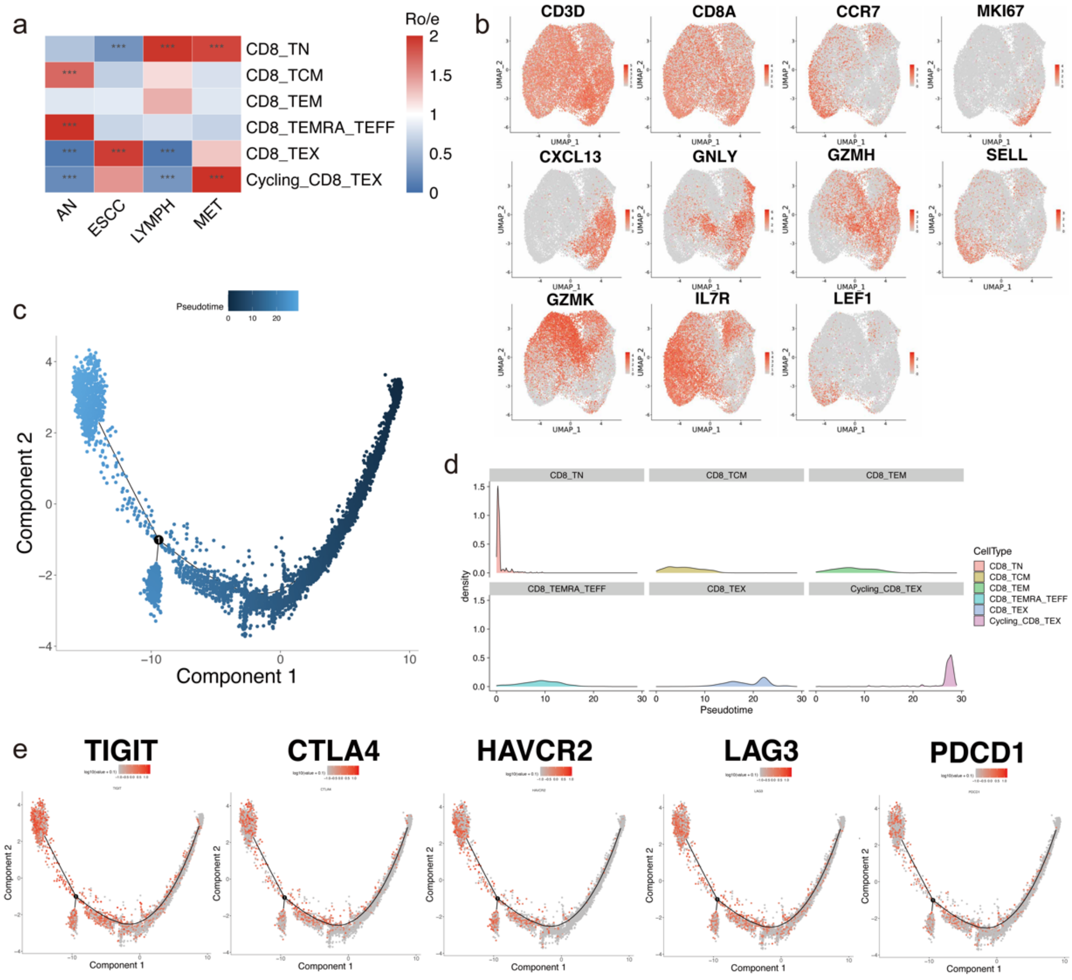

Supplementary Fig.3 Exhausted CD8$^+$ T cells in cycling status serve as an essential role in metastatic ESCC. a. Heatmap comparing the proportion of CD8$^+$ T cells among tumor samples, adjacent normal tissue, metastatic lymph nodes and non-metastatic lymph nodes. *P < 0.05; **P < 0.01; ***P < 0.001. b. UMAP plots showing the expression levels of key marker genes of each CD8$^+$ T cell subcluster. c. Trajectory of CD8$^+$ T cells along pseudotime in a two-dimensional space. Each single cell is represented by a point. d. The abundance of each CD8$^+$ T cell subcluster in the potential trajectory along pseudotime. e. The expression levels of immune checkpoint inhibitors in the trajectory of CD8$^+$ T cells along pseudotime in a two-dimensional space.

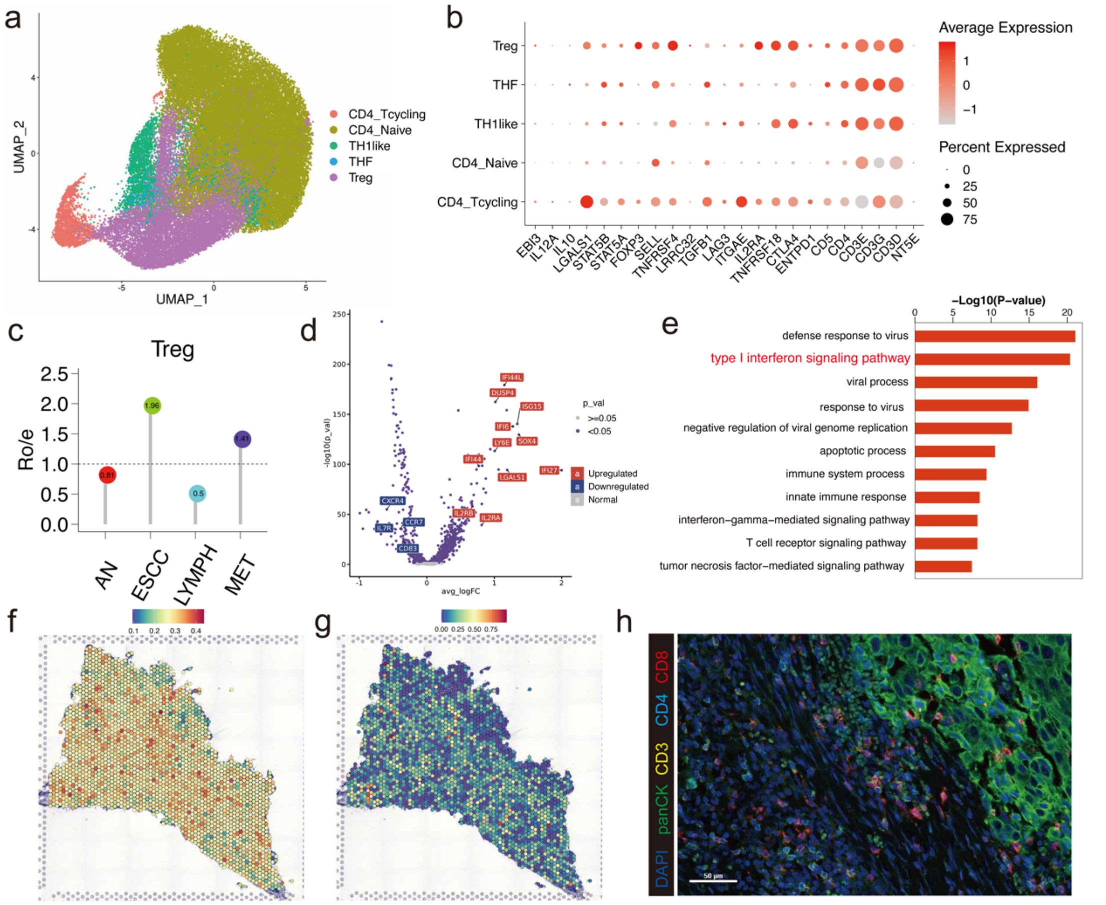

Supplementary Fig.4 Type-I interferons secreted by CD4^{+} Tregs may facilitate metastasis in ESCC. a. UMAP plots of CD4^{+} T cells grouped into 5 clusters. b. Bubble plot showing the key marker genes of each subcluster of CD4^{+} T cells. c. The relative proportion of CD4^{+} Treg among different samples. d. Volcano plot showing the differentially expressed genes of CD4^{+} Tregs between metastatic lymph nodes and lymph nodes. e. Biological processes enriched in the upregulated genes of CD4^{+} Treg obtained from metastatic lymph nodes. f. Spatial distribution of CD4^{+} Treg in the tumor site of one patient with metastatic lymph nodes. g. The deconvolution analysis showing the distribution of CD4^{+} Tregs of one patient with metastatic lymph nodes. h. The mIHC staining of CD3, CD4 and CD8 in the tumor site of one patient with metastatic lymph nodes. Scale bars: 50 μm.

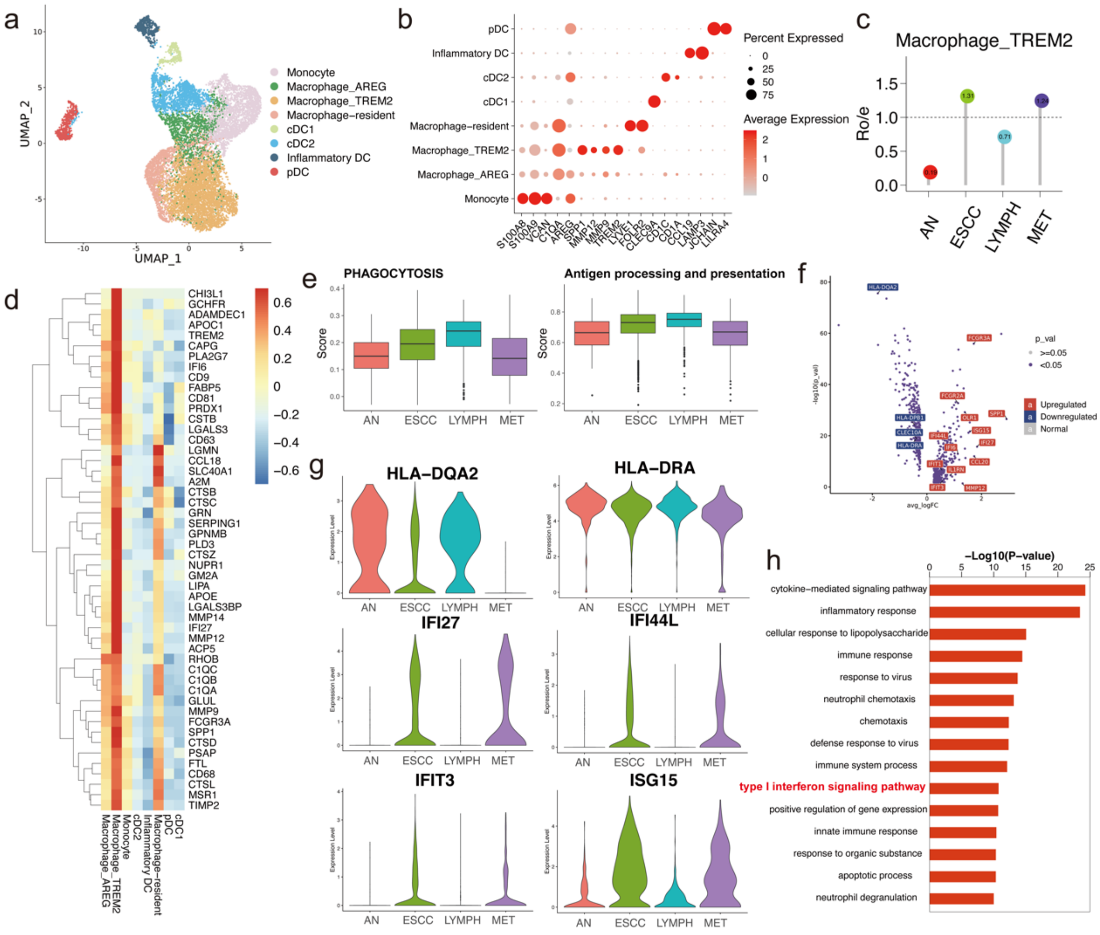

Supplementary Fig.5 Profiles and functions of myeloid cells in metastatic ESCC. a. UMAP plots of 13,122 myeloid cells grouped into 8 clusters from 29 samples of 12 patients, with each color coded by either main annotated cell type. b. Bubble plot showing the key marker genes of each subcluster of myeloid cells. c. The relative proportion of TREM2^{+} macrophages in tumor samples, adjuvant tissues, lymph nodes and metastatic lymph nodes. d. Heatmap depicting top genes highly expressed in TREM2^{+} macrophages. e. Comparison of specific biological processes of TREM2^{+} macrophages among different samples. f. Volcano plot depicting the differentially expressed genes in TREM2^{+} macrophages between lymph nodes and metastatic lymph nodes. g. Expression levels of specific genes in TREM2^{+} macrophages among different samples. h. Biological processes enriched in TREM2^{+} macrophages of metastatic lymph nodes.
